# Nanosensors based on LSPR are able to serologically differentiate dengue from Zika infections

**DOI:** 10.1038/s41598-020-68357-9

**Published:** 2020-07-09

**Authors:** Alice F. Versiani, Estefânia M. N. Martins, Lidia M. Andrade, Laura Cox, Glauco C. Pereira, Edel F. Barbosa-Stancioli, Mauricio L. Nogueira, Luiz O. Ladeira, Flávio G. da Fonseca

**Affiliations:** 1 Laboratório de Virologia Básica e Aplicada, Departamento de Microbiologia, Instituto de Ciências Biológicas, Universidade Federal de Minas Gerais, Belo Horizonte, MG Brazil; 2 Laboratório de Química de Nanoestruturas de Carbono, Centro de Desenvolvimento da Tecnologia Nuclear-CDTN/CNEN, Belo Horizonte, MG Brazil; 3 Laboratório de Nanomateriais, Departamento de Física, Universidade Federal de Minas Gerais, Belo Horizonte, MG Brazil; 4 Fundacao Ezequiel Dias, FUNED, Belo Horizonte, MG Brazil; 5 Laboratório de Pesquisa em Virologia, Departamento de Doenças Infecciosas e Parasitárias, Faculdade de Medicina de São José Do Rio Preto, São José do Rio Preto, SP Brazil; 6 NanoBioMedical Research Group, Departamento de Física, Universidade Federal de Minas Gerais, Belo Horizonte, MG Brazil; 7 Centro de Tecnologia de Vacinas, Universidade Federal de Minas Gerais, Belo Horizonte, MG Brazil; 8 Present Address: Laboratório de Pesquisa em Virologia, Departamento de Doenças Infecciosas e Parasitárias, Faculdade de Medicina de São José Do Rio Preto, São José do Rio Preto, SP Brazil

**Keywords:** Biosensors, Infectious-disease diagnostics

## Abstract

The *Flaviviridae* virus family was named after the *Yellow-fever virus*, and the latin term *flavi* means “of golden color”. Dengue, caused by *Dengue virus* (DENV), is one of the most important infectious diseases worldwide. A sensitive and differential diagnosis is crucial for patient management, especially due to the occurrence of serological cross-reactivity to other co-circulating flaviviruses. This became particularly important with the emergence of *Zika virus* (ZIKV) in areas were DENV seroprevalence was already high. We developed a sensitive and specific diagnostic test based on gold nanorods (GNR) functionalized with DENV proteins as nanosensors. These were able to detect as little as one picogram of anti-DENV monoclonal antibodies and highly diluted DENV-positive human sera. The nanosensors could differentiate DENV-positive sera from other flavivirus-infected patients, including ZIKV, and were even able to distinguish which DENV serotype infected individual patients. Readouts are obtained in ELISA-plate spectrophotometers without the need of specific devices.

## Introduction

Dengue is an arboviral infection that is endemic in countries of Asia, Oceania, the Americas, Africa, and the Caribbean. The US Centers for Disease Control and Prevention (CDC) estimates that about 40% of the world’s population live in regions where the risk of dengue transmission is high^1^. The last comprehensive study on global dengue burden has put the number of yearly infections in about 390 million^2^ and even though the study was published a few years ago the World Health Organization (WHO) still consider those as the most likely actual numbers^3,4^. *Dengue virus* (DENV)*,* the pathogen that causes dengue fever and other manifestations, is classified as part of the *Flavivirus* genus within the *Flaviviridae* family. The family was named after the *Yellow fever virus* (YFV) and the Latin particle *Flavi* means “of golden color”—a reference to the onset of jaundice observed in YFV-infected patients. Flaviviruses are enveloped viruses whose genome encodes just one *open reading frame* (ORF) that codifies a single polyprotein. During the virus replication cycle the polyprotein is cleaved in three structural and seven nonstructural polypeptides by virus-coded or cell proteases^5^. The DENV Envelope protein (DENV E) is an immunodominant polypeptide that is inserted into the virus envelope and exposed on the virus surface, mediating the adsorption to host cells and membrane fusion upon cell entry^6^.

There are four known DENV serotypes which are genetically and antigenically distinct, and each one is able to cause clinical manifestations ranging from asymptomatic infections to severe disease or even death^7,8^. DENV infections by any serotype induce protective immune responses against subsequent infections with the same serotype, whereas heterotypic secondary infections may lead to exacerbated viral multiplication and the development of severe disease^9–11^. The *Zika virus* (ZIKV) (a closely related flavivirus) emergence in areas where other flaviviruses circulate brought a significant burden to an already complicated scenario, in which affected countries must frequently cope with yellow fever, dengue and epidemics caused by other arboviruses. This is especially true after the recent ZIKV outbreaks in the Americas revealed an association of the infection to the occurrence of neurological malformations in fetuses from infected mothers and neurogenic demyelinating diseases such as the Guillain–Barre syndrome^12^.

Flaviviruses are known to remain viremic for a relatively short period of time during infection (typically 3–7 days after the appearance of symptoms, or longer in the case of pregnant women with ZIKV infections), and this narrow window complicates the detection of virus’ nucleic acids or antigens to confirm infections^13,14^. Therefore, serology continues to be the predominant diagnostic tool in terms of clinical practice, especially serology tests like immunofluorescence assay (IFA) and enzyme-linked immunosorbent assay (ELISA). Nonetheless, physicians and public health authorities must be aware of the high antigenic similarity among flaviviruses (e.g. 54–59% of amino acid sequence similarity between the DENV and ZIKV E proteins), which limits the use of serology-based tests to distinguish these infections due to intense antisera cross-reactivity between viruses^15,16^. Such limitations in the use of conventional diagnostic methods have driven the search for new diagnostic platforms, especially those able to deliver better sensitivity and specificity scores.

The upsurge of the Nanotechnology has induced the generation of many new materials that present potential to be used in association to antigens as diagnostic tools. Metallic gold nanoparticles (GNPs) are highly stable particles with features that make them very attractive in biological applications. Such features include attainable surface functionalization chemistry; capability to be synthesized in many different shapes; shape- and size-dependent optical and electronic characteristics; and many other pertinent properties^17^. One of the most important optical features of GNPs is the fact that when they are irradiated with light of specific frequencies this results in the collective oscillation of electrons in the particle surface. Such oscillation is named Localized Surface Plasmon Resonance (LSPR). This phenomenon happens when the oscillating electromagnetic field of the incident light interacts with electrons on the conduction band of the metal initiating their oscillation in resonance with the frequency of the light. As electrons oscillate, a charge separation between the free electrons and the ionic core on the metal occurs, exerting a restoring Coulomb force that causes the electrons to oscillate back and forth on the metallic surface, creating a dipole oscillation^18^.

When the plasmon frequency is of the same as the incident light, a resonance phenomenon occurs and results in a noticeable optical absorption. Thus, the coupling of the incident light and plasmon mode generates a sharp electric field on the surface of the metallic nanoparticles. In the spherical gold nanoparticles’ LSPR, the plasmon absorption frequency is independent of the excitation light polarization, with a peak in the 515–520 nm position, presenting low sensitivity for dielectric surface changes. Non-spherical nanoparticles like gold nanorods, however, present two LSPR modes, one transverse and another longitudinal, with incident light polarizations perpendicular and parallel to the axis of the rod, respectively. The transverse mode has low sensitivity to the dielectric surface changes, however, the longitudinal mode presents a strong polarization effect and high sensitivity to dielectric surface changes, making such particles responsive to the dielectric features of the surrounding environment^19^. Therefore, any modifications around the nanoparticle, including alterations in their surface, the solvent and particle aggregation, will determine changes in the electronic properties of the nanoparticle’s surface, resulting in alterations in the patterns of the absorption spectrum. All these optical features make GNPs useful in many different biological applications, particularly to detect molecules found in low concentrations on the environment^20^. Due to the high LSPR sensitivity, different LSPR-based biosensors have been reported recently, from cardiac biomarkers^21^ to bovine IgG detectors^22^, and more. Here, we describe the construction and testing of a Dengue GNR-based nanosensor in which the surface E protein of each DENV serotype was individually associated to GNRs. When tested against commercial antibodies and antisera from different flaviruses-infected patients the GNR sensors were able not only to detect the presence of antibodies in very low concentrations but were also able to distinguish DENV-infected individuals from seronegative ones and from those that were infected by other flaviviruses. Because antibody detection is achieved by evaluating shifts in the light absorption frequencies of the sensor, biosensing can be made with the use of regular spectrophotometers that are usually available in public health centers at remote and under-developed geographic areas where arboviruses so frequently circulate.

## Results

### Validation of the recombinant DENV 1–4 envelop proteins

**Figure 1:**
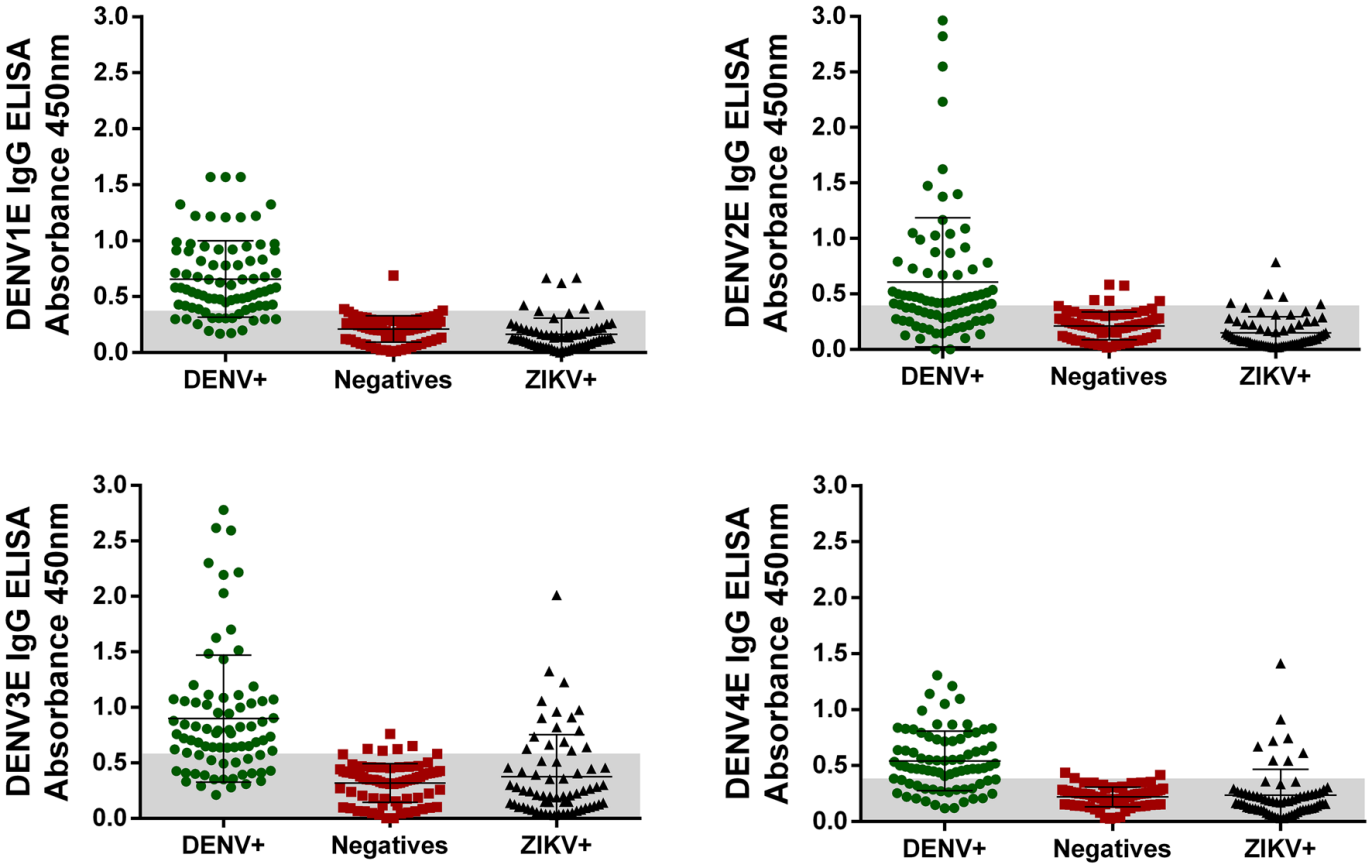
Evaluation of the diagnostic performance of the DENV recombinant proteins (DENV1E, top left; DENV2E, top right; DENV3E, bottom left; and DENV4E, bottom right) by an in-house ELISA test. Each green dot represents the serum sample of a DENV IgG-positive subject; each red dot represents the serum of an arbovirus-seronegative subject; and each black dot represents serum samples from ZIKV seropositive subjects. The gray area represents the calculated cutoff for each protein. The mean absorbance value of each group is represented by the larger horizontal lines on each row. Standard deviations are also represented.

In order to build a LSPR-GNR-based sensor, we generated recombinant proteins based on the Envelope protein coding genes of each of the four DENV serotypes. The expression and purification of the different recombinant E proteins (named DENV1E, DENV2E, DENV3E and DENV4E) were evaluated by SDS-PAGE (Suppl Fig. 1A). The antigenic potential of these recombinant proteins (of approx. 50 kDa each) was determined by Western blot using a specific monoclonal antibody against *Dengue virus* (Suppl Fig. 1B). Identical blots were also probed with an antibody against the histidine tail fragment to further confirm the production and purification of the recombinant proteins (Suppl Fig. 1C). To analyze the diagnostic potential of these proteins, we developed an in-house ELISA platform and tested it against DENV-positive sera, ZIKV-positive sera and flavivirus-negative sera (Fig. 1) (characterization of the sera banks used here can be seen in Suppl Tables 1 and 2). The ELISA tests were able to efficiently distinguish DENV-negative sera from the sera of DENV-infected patients (comparing DENV+ and negative rows in each graph). Nevertheless, sensitivities were not robust as many patients (individual dots) still fell below the cutoff area, especially for DENVE2 and DENVE4 proteins. The global sensitivity varied from 50.9 to 80.2% whilst the specificity varied from 85.5 to 93.7%. The specificity values represent the ability of the ELISA test to distinguish dengue-infected patients from Zika seropositive individuals, in this case. All diagnostic performances and parameters of this test can be accessed at Suppl Table 3. Definitions as well as how sensitivity (TSe) and specificity (TSp) were calculated are presented in the methods’ session.

### Construction and characterization of the LSPR-DENV nanosensor

**Figure 2:**
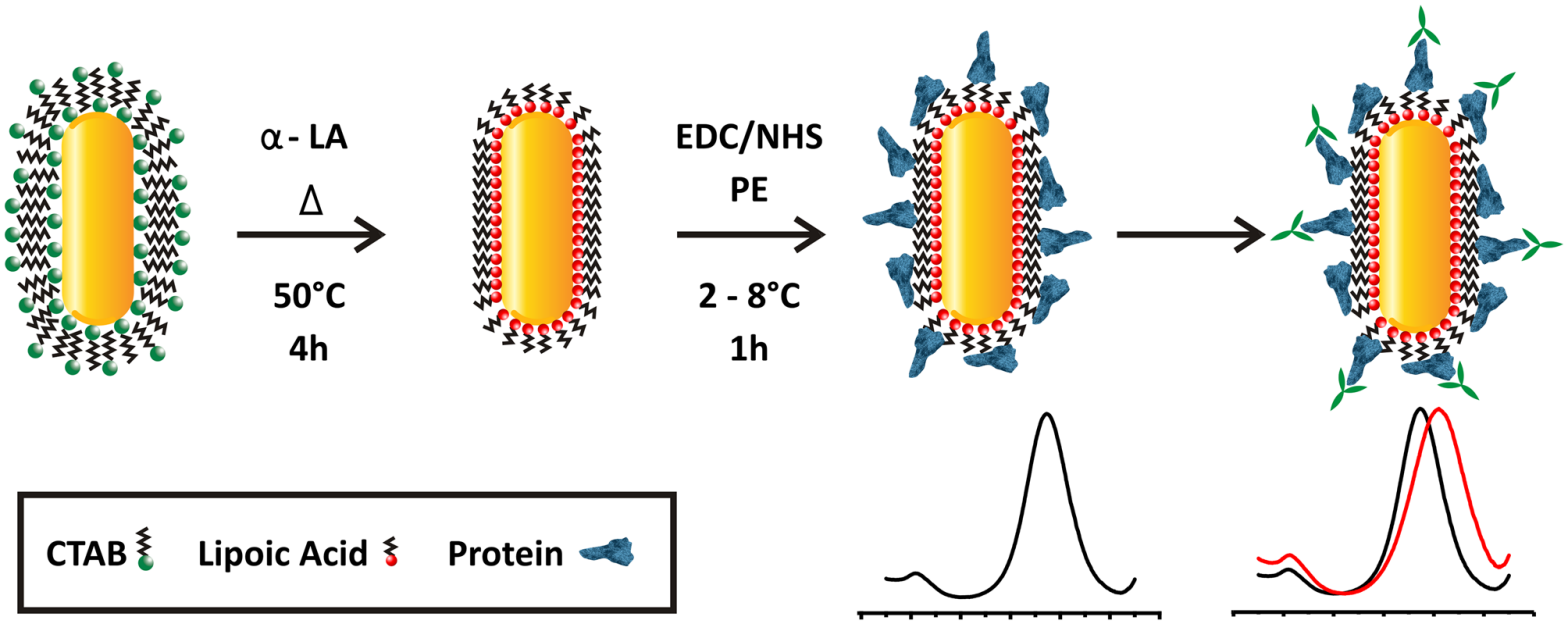
DENV LSPR-Nanosensor construction. The gold nanorods were primarily linked to α-lipoic acid through interaction with the molecule’s thiol group. Subsequently, EDC and NHS reagents were added to drive and stabilize substitution reactions through which recombinant proteins were covalently linked to the α-lipoic acid on the surface of the GNRs. The protein-decorated GNRs were used as biosensors in which specific antibodies bind to their cognate proteins on the GNR surface. Antibody binding disrupts the LSPR pattern on GNRs which can result on a shift of the emitted spectrum (bottom graphs). *α-LA* α-lipoic acid, *EDC* 1-ethyl-3- (3-dimethylaminopropyl) carbodiimide, *NHS* N-hydroxysuccinimide.

We successfully functionalized GNRs with each protein—DENV1E to DENV4E—using established protocols with modifications. The functionalization strategy, depicted in Fig. 2, consists on the attachment of proteins to the gold nanorods by covalently coupling the amine terminal functional groups of polypeptides to so called capping agents, a process well described in the literature^23–25^. We used a reduced α-Lipoic acid (DHLA) as capping agent and sulfo-NHS (*N*-hydroxysulfosuccinimide) and EDC (1-Ethyl-3-(3-Dimethylaminopropyl)carbodiimide) as intermediate chemicals—the later reagents direct the formation of an ester bridge between the protein and the DHLA, resulting in the covalent interaction among these molecules. The choice of the reduced α-Lipoic acid is related not only to the its proper function as a linker to protein binding, but also as a strategy to remove the CTAB (cetyltrimethylammonium bromide) monolayer covering the nanoparticles in solution. Because CTAB is related to the stability of GNRs in solution^26^, it was important to analyze whether CTAB-stripped and DHLA functionalized GNRs were stable. Indeed, GNR-DHLA particles were evaluated for 16 months using UV–Vis spectroscopy and no major changes at the observed light absorbance spectra was observed (Suppl Fig. 2). Consistently, there was no color change or precipitations in the stored solutions (not shown).

**Figure 3:**
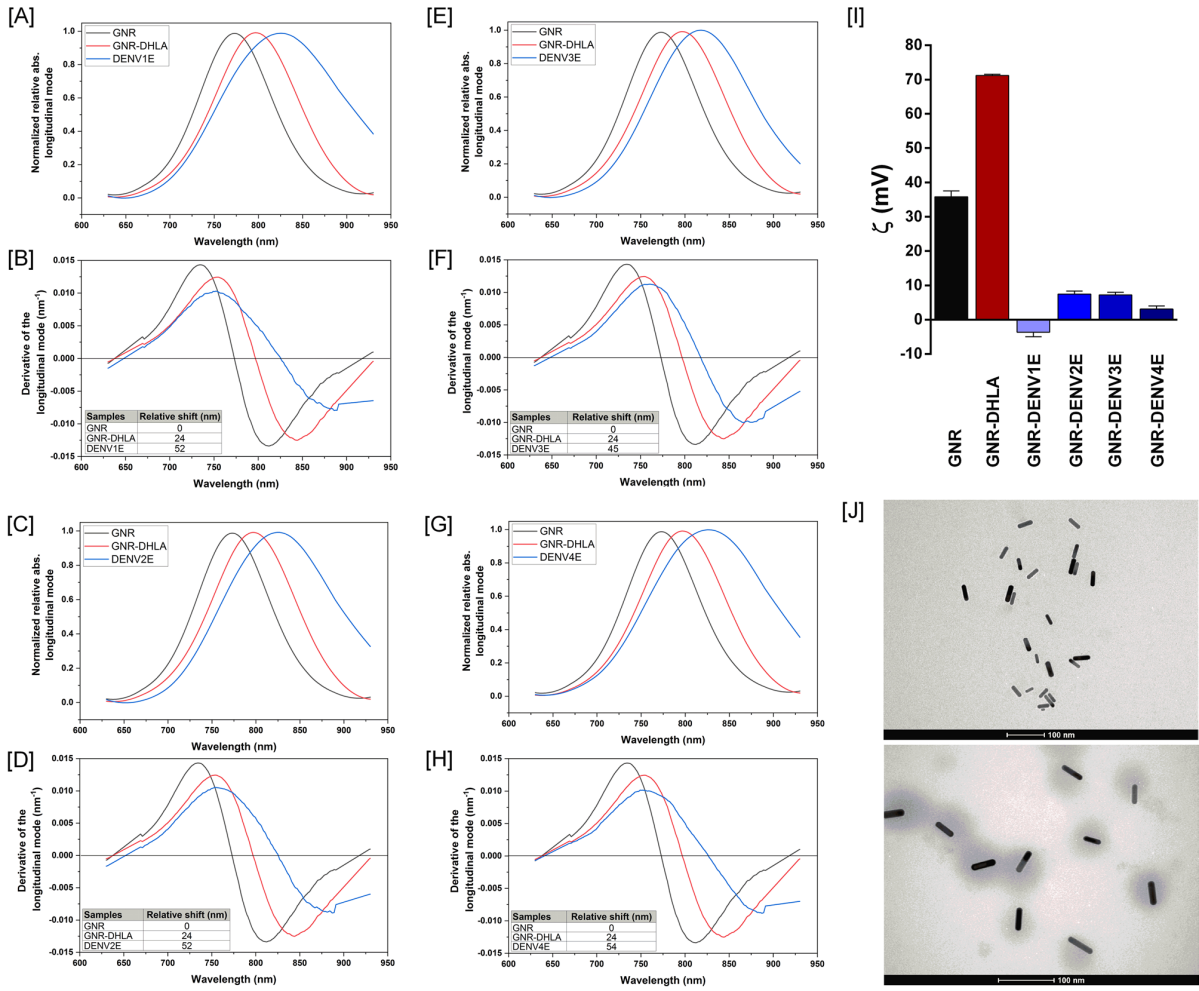
DENV LSPR-Nanosensors characterization. (**A**,**C**,**E**,**G**) UV–Vis-NIR spectroscopy measurements. Black line represents the pristine GNR spectrum; red line represents the spectrum shift (24 nm) after α-DHLA interaction; and blue line represents the GNR-protein spectrum: (**B**,**D**,**F**,**H**) Derivative calculation from the LSPR signal measured from the samples, where the region of zero (derivative axis) represents the peak of the longitudinal mode. Considering the pristine GNR spectrum as a reference, relative calculated shifts after reaction to each DENVE proteins are (**B**) 52 nm to DENV1E, (**D**) 52 nm to DENV2E, (**F**) 45 nm to DENV3E, and (**H**) 54 nm to DENV4E. (**I**) Zeta potential measurements of pristine GNR (black), GNR-DHLA (red) and proteins (blue) showing the variability of GNR total charge after each functionalization step. (**J**) TEM images before (top) and after (bottom) functionalization process. After the functionalization process, an “amorphous substance” is capping the GNR. Panels are representative of all four generated sensors. Samples from sera banks depicted on Tables S1 and S2 (supplementary material) were used in this experiment.

The efficiency of the GNR-DENVE functionalization was confirmed by three different approaches. First, we performed UV–Vis measures before, during and after the functionalization process to determine electrical changes on the GNR surface. The LSPR spectrum shifts observed after chemicals (red lines) and protein binding (blue lines) indicate the successful GNR functionalization with each DENVE protein (Fig. 3A,C,E,G). To determine the correct plasmon shift, each LSPR curve was plotted with the correspondent calculus of the X axis intercept of the derivative of the Gaussian peak (Fig. 3B,D,F,H). Second, both pristine GNRs and GNR-DENVE nanosensors were evaluated through Zeta potential analysis, and the GNR total charge modifications after each functionalization step confirmed the correct association of the GNRs to the recombinant DENVE proteins (Fig. 3I). At last, transmission electron microscopy (TEM) was used to observe surface structural modifications during GNR functionalization. The TEM images show the overall GNR structure before functionalization (Fig. 3J, top panel) and the deposition of an amorphous layer capping the GNR after the functionalization with DENVE (Fig. 3F, bottom panel). Taking together, these results indicate the effective interaction between the DENVE proteins and GNRs, and the correct generation of the all four DENV serotype-specific nanosensors. All tests described above are regularly used in the characterization of functionalized GNRs^27,28^.

### Testing of the LSPR-DENVE nanosensors using monoclonal antibodies and human clinical samples

**Figure 4:**
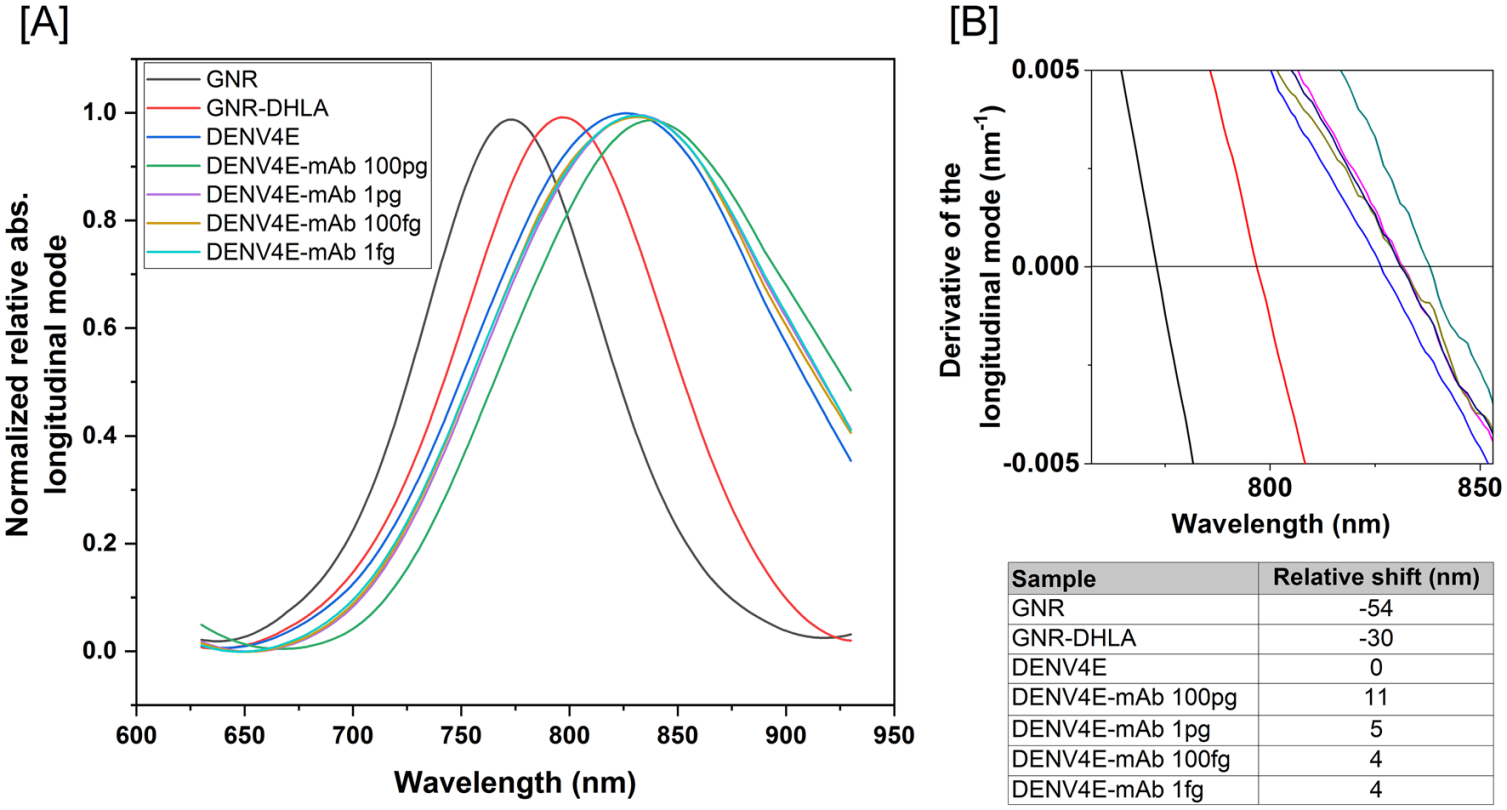
Representative evaluation of the LSPR-GNR-Nanosensors’ ability to detect low amounts of specific antibodies using different concentrations of a dengue monoclonal antibody. The nanosensor were tested with different concentrations of monoclonal antibody (commercial, DENV-specific), varying from nanograms (ng) to femtograms (fg). All tested nanosensors were capable to detect antibodies at picograms range—as shown here with GNR-DENV4E. (**A**) Absorbance spectra for pristine gold nanorods (GNR), nanorods capped with lipoic acid (GNR-DHLA) and the completed sensor (DENV4E) are shown. (**B**) Derivative calculation from the LSPR signal measured from the samples. The region of zero (derivative axis) and 750–850 nm (wavelength axis) represents the peak of the longitudinal mode. The equipment wavelength accuracy is ± 3 nm, therefore, for those tests only shifts equal or higher than 5 nm were considered significant. Wavelength shifts for different monoclonal antibodies’ dilutions are shown in (**B**). Shifts depicted at the inset table are related to the sensor with no sera spectrum (DENV4E).

**Figure 5:**
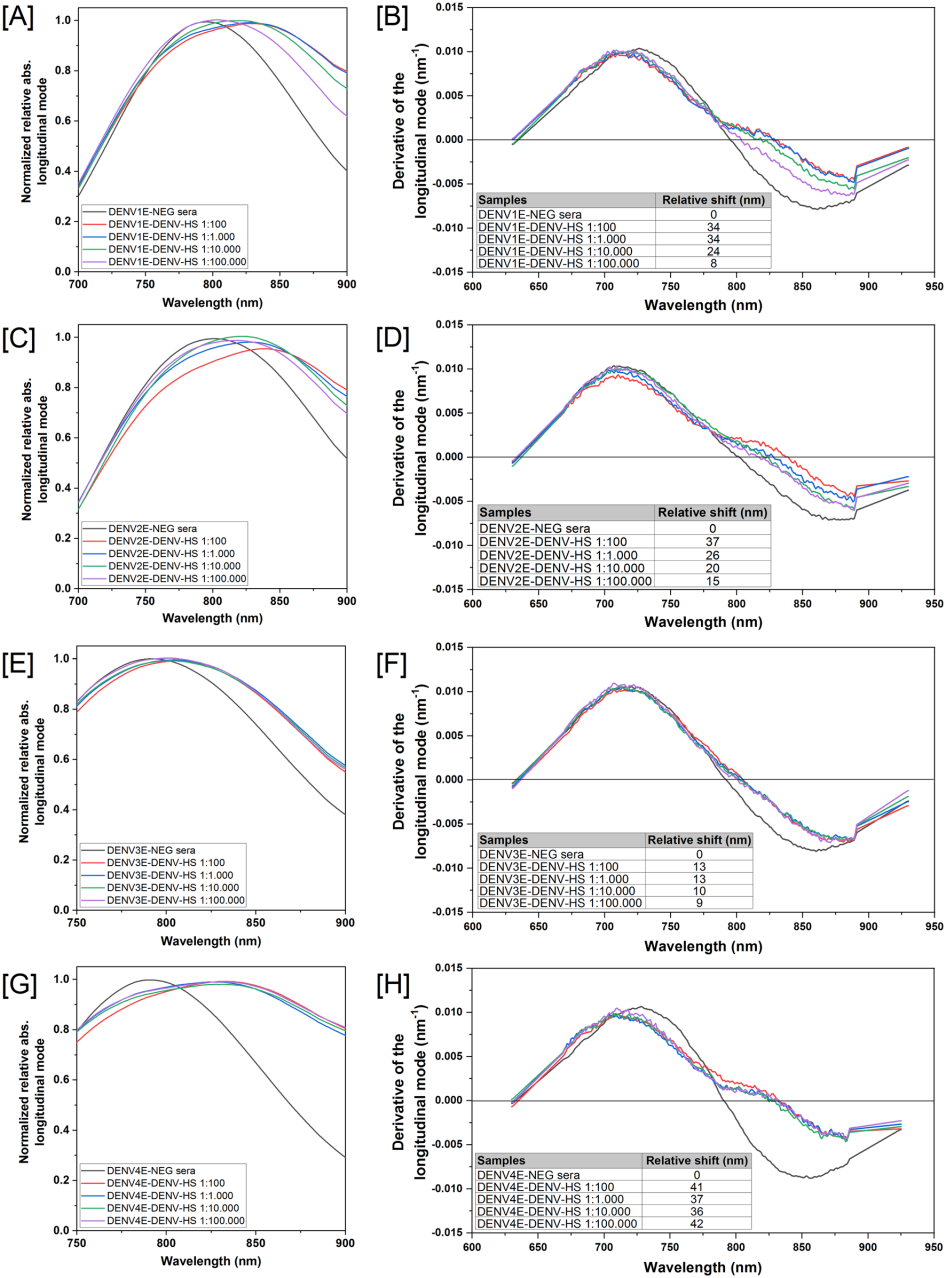
Evaluation of the LSPR-GNR-Nanosensors limit of detection using a serially diluted pool of DENV-positive human antisera. The pooled sera were diluted in 1:10 to 1:100,000 ratios and added to each specific sensor: (**A**,**B**) GNR-DENV1E; (**C**,**D**) GNR-DENV2E; (**E**,**F**) GNR-DENV3E; and (**G**,**H**) GNR-DENV4E. Reads were obtained between 750 and 900 nm wavelength range (graphs in **A**,**C**,**E**,**G**). Relative shifts on the region of zero of the derivative curves for different sera dilutions are shown below each graph (graphs in **B**,**D**,**F**,**H**). The equipment wavelength accuracy is ± 3 nm and shifts equal or higher than 5 nm were considered significant. Shifts are related to the sensor added with dengue-negative sera (DENVE-NEG sera). Samples from the sera bank depicted on Table S2 (supplementary material) were used in this experiment.

Having constructed the functionalized GNR sensors, we evaluated their sensitivity and specificity through interactions with diluted samples from a panel of antibodies and sera, including dengue monoclonal antibodies, sera from dengue seropositive or seronegative human individuals, as well as sera from patients that are positive for other flaviviruses. The first test was conducted to confirm the correct interaction between proteins and the nanoparticles and the maintenance of available antibody binding sites. More importantly, having a known concentration, anti-Dengue commercial monoclonal antibodies were used to determine the minimum anti-Dengue antibody amounts that can be detected by the sensors. establish limits of detection for the nanosensors. Commercial antibodies of the same brand and batch were used in all experiments. Antibodies were tenfold diluted until concentrations of 1 fg and incubated with the nanosensors. Figure 4 shows the spectral shifts in the GNR-DENV4E sensor for the final four dilutions in relation to the sensor with no sera added. The nanosensor was able to detect as low as 1 pg of anti-dengue antibodies with a 5 nm wavelength shift in the absorbance spectra in relation to the sensor with no antibodies added. Similar shifts were observed for the three other sensors (data not shown). Likewise, when pooled sera from dengue immune individuals were tested in our nanosensing platforms, dilutions of up to 1:100,000 induced significant wavelength shifts in the observed absorption plasmon bands, especially on the longitudinal bands (Fig. 5A,C,E,G). The GNR-DENV4E sensor presented the best performance, with a shift of 42 nm in the 1:100,000 sera dilution range in relation to the DENV-negative sera (Fig. 5H). Nonetheless, the other three sensors performed similarly, with relative shifts between 8 and 15 nm in relation to the DENV-negative control at the highest sera dilutions (Fig. 5B,D, F).

**Figure 6:**
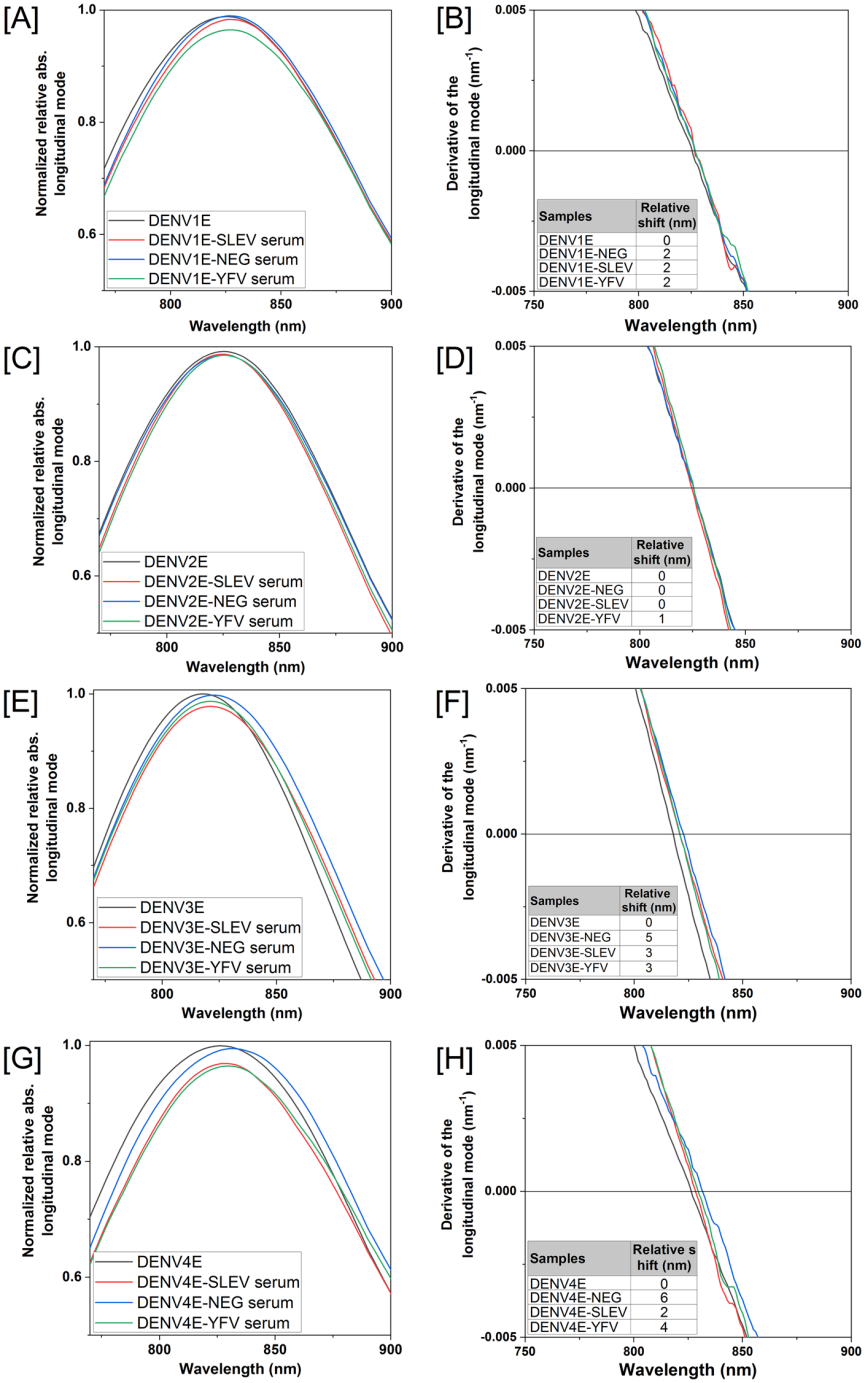
Specificity of the DENV LSPR-Nanosensors. Individual sensors were incubated with human flavivirus-negative serum (blue lines), human Saint Louis encephalitis virus-positive serum (SLEV, red lines), or human Yellow Fever virus-positive serum (YFV, green lines). Specific sera were pooled and diluted in 1:10,000 rations. (**A**,**B**) Nanosensor GNR-DENV1E, (**C**,**D**) Nanosensor GNR-DENV2E, (**E**,**F**) Nanosensor GNR-DEN3E and (**G**,**H**) Nanosensor GNR-DENV4E. Reads were obtained between 750 and 900 nm wavelength range (graphs in **A**,**C**,**E**,**G**). Relative shifts on the region of zero of the derivative curves for each serum are shown below each graph (graphs in **B**,**D**,**F**,**H**). The equipment wavelength accuracy is ± 3 nm and shifts equal or higher than 5 nm were considered significant. Shifts are related to the sensors suspended in 10 mM phosphate buffer with no sera added. Samples from sera banks depicted on Tables S1 and S2 (supplementary material) were used in this experiment.

**Figure 7:**
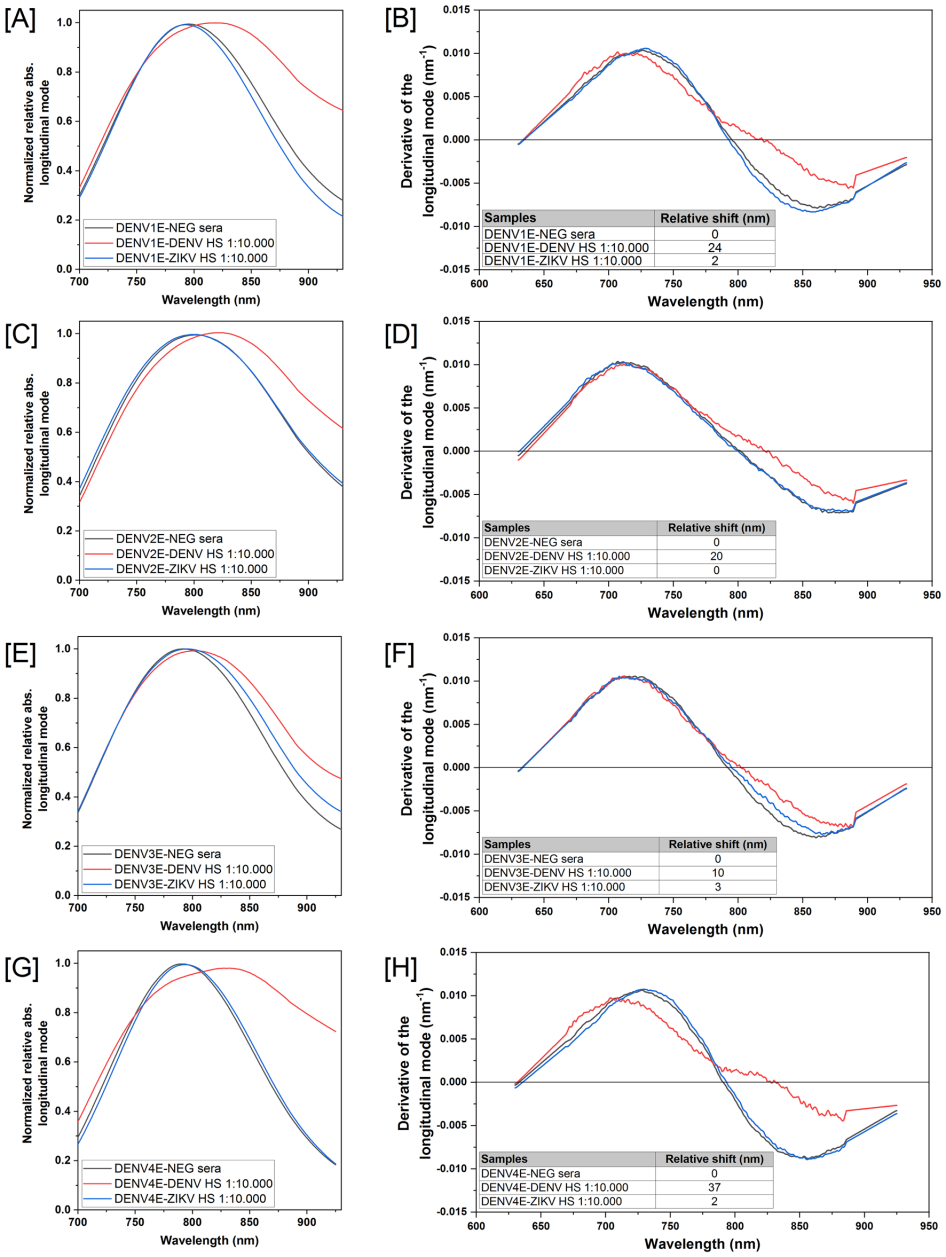
Evaluation of the LSPR-Nanosensor capability to distinguish DENV from ZIKV infections. Human Dengue virus-positive sera (DENV—red lines) and Zika virus-positive sera (ZKV—blue lines) were pooled and diluted in 1:10,000 ratios. Pools were individually added to each sensor GNR-DENV1E (**A**,**B**); GNR-DENV2E (**C**,**D**); GNR-DENV3E (**E**,**F**) or GNR-DENV4E (**G**,**H**). Flavivirus-negative pooled sera were used as controls (black lines). Reads were obtained between 700 and 950 nm wavelength range (graphs in **A**,**C**,**E**,**G**). Relative shifts on the region of zero of the derivative curves for each serum are shown below each graph (graphs in **B**,**D**,**F**,**H**). The equipment wavelength accuracy is ± 3 nm and shifts equal or higher than 5 nm were considered significant. Shifts are related to the sensor added with dengue-negative sera (DENVE-NEG sera). Samples from the sera bank depicted on Table S2 (supplementary material) were used in this experiment.

When it comes to flaviviruses’ diagnostics, however, the specificity of a given test is as important as its sensitivity, because cross-reactions among related virus species and antibodies are a real problem. To evaluate the specificity of the sensors, we tested them with sera from dengue-negative individuals as well as from individuals that were seropositive for other flaviviruses, including *Saint Louis encephalitis virus* (SLEV) and YFV. In almost all cases, we detected no significant wavelength shifts (above 5 nm) during the UV–Vis spectroscopy evaluation (Fig. 6). Significant spectra shifts of were observed only when DENV-negative sera pools were added to DENV3E and DENV4E sensors (Fig. 6F,H). Nonetheless, as mentioned before, the global emergence of ZIKV and the difficulties to serologically discriminate ZIKV infections from DENV infections added more pressure over the Public Health systems in affected countries. Therefore, we tested the ability of our GNR sensors to discriminate dengue IgM-seropositive individuals from those that were recently infected with ZIKV employing a well-characterized human sera bank (Suppl Table 1). We observed wavelength shifts equal or higher than 20 nm for dengue sensors DENV1, 2 and 4 using 1:10,000 sera dilution ratios of dengue-positive samples, whereas same dilution ratios from ZIKV-infected patients produced between zero and 2 nm in wavelength shifts (Fig. 7). All shifts were observed in relation to sensors tested with flavivirus-negative sera pools. Nanosensors GNR-DENV1E, GNR-DENV2E and GNR-DENV4E showed no significant cross-reactivity with ZIKV sera and presented wide shifts when tested against the dengue-positive sera (24 nm, 20 nm, and 37 nm, respectively—Fig. 7B,D,F,H). Nanosensor GNR-DENV3E presented a narrow shift in comparison to the other sensors when tested to the serotype-specific sera (10 nm) but with no significant cross-reactivity with ZIKV sera (Fig. 7F).

**Figure 8:**
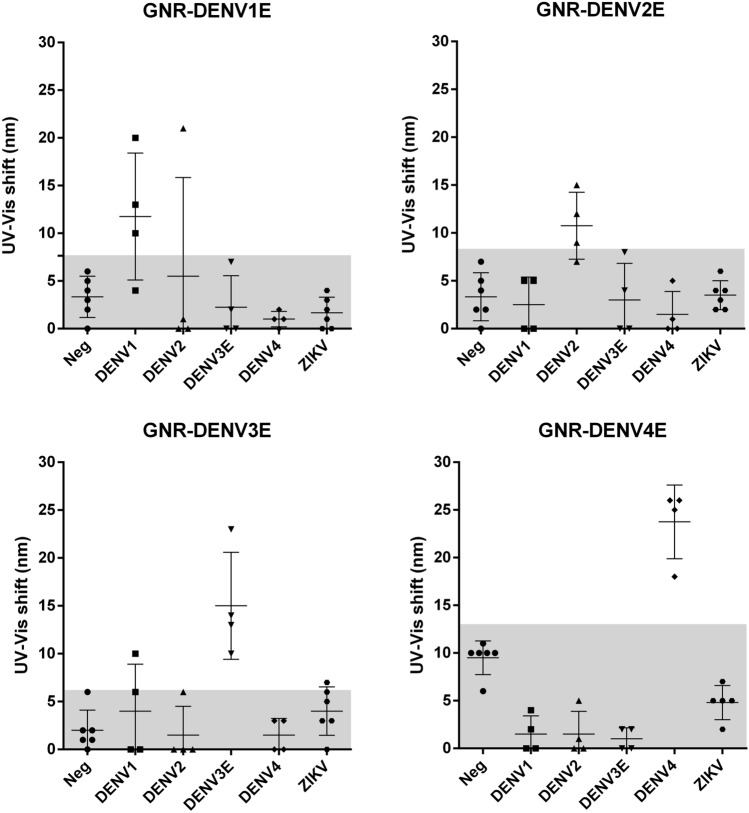
Evaluation of the LSPR-Nanosensor capability to distinguish DENV specific serotypes. Serotype specific Dengue virus-positive antisera, Zika virus-positive antisera and flavivirus-negative antisera were evaluated by molecular, serologic and PRNT analyses. Each individual serum was diluted in a 1:100,000 ration and incubated with each sensor (GNR-DENV1E; GNR-DENV2E; GNR-DENV3E; or GNR-DENV4E). The Y axis in each graph represents the spectrum shift in nm; each position on the X axis represent either serotype specific dengue-positive individuals (DENV1, 2, 3 and 4), zika-positive individuals (ZIKV); or flavivirus-negative individuals (Neg). Dots represent individual serum. The gray area represents the cutoff value for each individual sensor, calculated based on the negative interaction background. The mean absorbance value of each group is represented by the larger horizontal lines on each row. Standard deviations are also represented. Samples from the sera bank depicted on Table S1 (supplementary material) were used in this experiment.

Having established our sensors’ ability to serologically distinguish DENV from ZIKV, SLEV and YFV infections, we decided to further evaluate them by testing their ability to serologically distinguish infections caused by even more closely related viruses: in this case, infections caused by each one of the four DENV serotypes. Each individual nanosensor (GNR-DENV1E, GNR-DENV2E, GNR-DENV3E and GNR-DENV4E) was incubated with diluted sera preparations from patients that were specifically seropositive for DENV1, DENV2, DENV3, DENV4 or ZIKV infection, as well as with flavivirus-negative sera (Fig. 8). The UV–Vis spectroscopy analyses revealed that each specific nanosensor was capable to identify most of the correlated serotype-specific samples and distinguish them from patients infected with non-correlated serotypes. The GNR-DENV4E sensor, for instance, performed flawlessly, with no cross-reactivity to the other DENV serotypes or ZIKV above the determined cutoff value. Other sensors performed similarly, although not with the same accuracy as the GNR-DENV4E sensor. A compiled dataset of the wavelength relative shift values for each sensor in relation to the tested sera is presented on Suppl. Table 4. The overall evaluation of the diagnostic performance of the nanosensors as DENV serotype-specific tools is shown on Table 1. All sensors presented robust diagnostic parameters, varying from 75 to 100% of sensitivity and 88.2–100% of specificity in relation to their ability to identify a correlated DENV-positive serum sample.

**Table 1:** Diagnostic performance measurements for the LSPR-Nanosensor GNR-DENV1E, GNR-DENV2E, GNR-DENV3E and GNR-DENV4E.

Parameters	GNR-DENV1E	GNR-DENV2E	GNR-DENV3E	GNR-DENV4E
Cutoff	7.65	8.34	6.20	13.02
TSe (%)	75	100	100	100
TSp (%)	94.1	94.4	88.2	100
PPV (%)	75	75	66.7	100
NPV (%)	94.1	100	100	100
AC (%)	90.5	95.2	90.5	100
TP	3	3	4	4
TN	16	17	15	17
FP	1	1	2	0
FN	1	0	0	0

*TSe* total sensitivity, *TSp* total specificity, *PPV* positive predictive value, *NPV* negative predictive value, *AC* accuracy, *TP* true positive, *TN* true negative, *FP* false positive, *FN* false negative.

## Discussion

Diagnosing DENV infections is a high priority in countries affected by annual epidemics of dengue fever. The correct diagnostic is essential for patient managing and prognostic as there are no specific antiviral drugs to treat the infection. Depending on the observed clinical developments, the infected patient may be released for home care; treated as an outpatient; or immediately admitted as an inpatient for emergency medical care. In all cases, laboratory confirmation of viral infection is crucial to the decision-making. Nonetheless, DENV infection diagnostic is no easy task, especially considering that tropical regions where dengue is most prevalent are also plagued by the circulation of other arboviruses causing diseases in which clinical manifestations are hardly distinguishable from dengue^29,30^. To complicate matters, some of these arboviruses, such as ZIKV and YFV, are closely relatives of DENV and extensive serological cross-reactions among them have been largely documented^31–34^. Genetic approaches and the Plaque Reduction Neutralizing Test (PRNT) are efficient methods to distinguish infections caused by different arboviruses. Nonetheless, these are labor-intensive, time-consuming methods not always available in the regions where they are necessary^35^. In addition, the mass application of those methods during an epidemic is virtually impossible, making more traditional serological diagnostic approaches, like ELISA and immunochromatography, the obvious and feasible choices. In these cases, however, cross-reactivity becomes an issue. In such scenario, nanostructured sensors become an attractive alternative due to their potential sensitivity and specificity aspects.

The choice for the DENV E protein as the sensor’s probing component is based on the fact that after an infection the majority of all antibody species produced against DENV recognize epitopes that are distributed on the virus’ surface proteins, either E or M, whereas a lesser proportion of these antibodies recognizes the NS1 non-structural protein^36^. Overall, serotype cross-reactive anti-E antibodies constitute the bulk of antibody responses elicited on a DENV-infected patient, followed by invariably smaller amounts of anti-(pr)M and anti-NS1^36, 37^. Conversely, any anti-DENV vaccine design must certainly include the E protein, as it is the main target of neutralizing antibodies which, despite some recent scrutiny, are still viewed as important correlates of immunological protection against the infection^36,38–40^. We produced truncated versions of the proteins in which the transmembrane C-terminus of each polypeptide was removed to improve the stability of the product. It has been shown by us and others that such modification causes no disturbance to the correct folding of the DENV E protein^41,42^. Nonetheless, bacteria-made DENV E recombinant proteins have been viewed as poor immunogens^43^, mostly because prokaryotic cells do not include post-translational modifications to the produced proteins, including the addition of carbohydrates to the polypeptide backbone. Indeed, most antibodies directed against the DENV E protein are thought to recognize discontinuous epitopes whereas only a few recognize linear epitopes^44,45^, and the presence of carbohydrates sterically influences the selection of discontinuous epitopes^46^. In fact, when we used our soluble recombinant E proteins in ELISA protocols to identify DENV-infected patients and distinguish them from either non-infected or ZIKA-infected patients we obtained mixed results. The DENV1E and DENV3E proteins were able to identify most DENV immune patients in our sera bank, distinguishing them from DENV-negative subjects and ZIKV immune individuals, although a few dengue-positive individuals were distributed bellow the cutoff area and some ZIKV-positive subjects were placed above it (Fig. 1, left panels). As for DENV2E and DENV4E ELISAs, however, identification of DENV immune individuals and distinction from the other groups were quite poor (Fig. 1, right panels). Nonetheless, all proteins could be efficiently recognized in Western-blot experiments using an anti-DENV commercial monoclonal antibody (Suppl Fig. 1). In the face of such inconclusive results and considering the economic advantages of having recombinant proteins produced in prokaryotic cells, we asked whether the construction of more sensitive diagnostic sensors could significantly enhance the diagnostic potential of the bacteria-made DENV1E to DENV4E recombinant proteins.

We constructed four GNR-based sensors in which recombinant E proteins of each DENV serotype were covalently linked to the surface of the rods employing reduced α-Lipoic acid as CTAB disperser and binding agent between the gold surface and the recombinant proteins. We monitored the correct construction of the sensors employing methods that are traditionally used for this purpose, including UV–Vis spectroscopy, TEM and Zeta potential measurements^27,28^. Having established that the nanosensors were successfully obtained, we individually tested the ability of each DENV serotype-specific sensor in recognizing correlate anti-sera while avoiding excessive cross-reactions with sera from individuals infected with other related flaviviruses, including SLEV, YFV and ZIKV. To that end, we took advantage of the LSPR property of gold nanoparticles and evaluated shifts at the observed light absorbance spectra when sensors were exposed to antibodies and sera. Our results indicated that all sensors were both highly sensitive and specific, with spectra shifts equal or higher than 5 nm when very low anti-DENV antibody amounts (Fig. 4) or highly diluted DENV-positive sera samples (Fig. 5) were added. On the other hand, incubation with sera from YFV- or SLEV-infected individuals produced spectra shifts that were consistently bellow the 5 nm cutoff limit in the spectra shift (Fig. 6). Indeed, according to Yu and Irudayaraj^47^, wavelength shifts as little as 5 nm are significant in LSPR-GNR systems, and low limits of detection using LSPR sensors as the ones described here have been documented for other nanosensing probes, especially when high affinity pairs—like antibody-to-antigen—are tested ^47–50^.

A especial attention was given to the ability of the described sensors to segregate DENV-infected patients from ZKV-infected ones. As demonstrated in Fig. 7, the GNR-DENV sensors were efficiently able to distinguish DENV-positive sera from ZIKV-positive samples. As mentioned before, ZIKV is a clinically relevant flavivirus that has been associated with important neurological malformations and stillbirths in women infected during pregnancy^12^. Nonetheless, the vast majority of infections in healthy adults result either on the absence of disease or in a clinical syndrome that is practically indistinguishable from dengue fever^51^. In addition, the serological differentiation between ZIKV and DENV infections is complicated by extensive cross-reactivity between these two viral agents because of their genetic proximity^31–34^. Therefore, traditional diagnostic methods based in antibody detection are severely compromised when it comes to distinguish patients that were infected with one virus or the other^51–53^ and, as a result, the PRNT remains as the gold standard for ZIKV diagnosis. Nonetheless, adding to the fact that the method is labor-intensive and not universally available, it has been suggested that even PRNTs may culminate in dubious results when applied to the sera of patients that have been infected with other flavivirus prior to ZIKV exposition, which is a common reality in the geographical areas where ZIKV circulates^35^. Molecular tests that detect the presence of ZIKV RNA are a viable and highly specific alternative; however, viremia is notably short during this virus’ infection^54^, leaving serology as a needed ZIKV diagnostic tool, despite its limitations. The development of sensors that could improve serological specificity and differentiate DENV from ZIKV has been described, including a microsphere immunoassay employing Luminex technology^55^ and electrochemical impedance spectroscopy coupled to square wave voltammetry^56^. However, the production of a nanosensor that is not dependent on specific and not thoroughly available reading equipment, as the one presented here, could be a real breakthrough for medical personal and public health authorities dealing with the problem in areas with limited access to highly specific technologies.

Although DENV and ZIKV are not in the same serocomplex within the *Flaviviridae* family, they share approximately 53–57% amino acid sequence identity in their E proteins, to which most abundant and neutralizing antibodies are elicited during infection^57^. The four DENV serotypes, on the other hand, share 63–78% amino acid identity in their E proteins^58^, enough to confer short-lived cross protection in secondary infections caused by a different DENV serotype. Due to this intense antigenic resemblance, the serologic differentiation of DENV serotypes during infections is considered impracticable. Nevertheless, when we tested highly diluted sera samples from DENV-seropositive individuals, each sensor was able to specifically recognize the correspondent anti-sera with accuracy (Fig. 8). Importantly, the human sera used to validate this aspect of the sensor were characterized by immunochromatography, ELISA and PRNT, adding the necessary confidence to the obtained results^35^. Although some samples were apparently identified by the non-correlated sensor on panels GNR-DENV1E, GNR-DENV2E and GNR-DENV3E (single dots on rows DENV2, DENV3 and DENV1, respectively), we considered these as outliers. Indeed, despite the outliers, the mean values (larger horizontal lines in each row) for the non-correlated samples on panels GNR-DENV2E and GNR-DENV3E remained within the shaded cutoff area. Moreover, it is not possible to rule out that these specific outlier patients have been previously exposed to a different DENV serotype, and in that case the sensors could have picked up this serological signal. To our knowledge, this is the first description of a serological method capable to identify efficiently which DENV serotype was responsible for the latest (primary or secondary) infection based solely on the serum sample of a given patient.

There is no doubt that nanotechnology has the disruptive power to affect our way of life in many ways, being incorporated from the concrete in the walls of our houses, in the micro-circuits of the TV remote, to the quality assessment of the food we eat. When it comes to biotechnology, the possibility of using nanomaterials to different ends is practically unlimited. Biosensors are amongst the most promising of all nanomaterial applications, bringing cheaper, more sensitive and more reliable detection platforms for an enormous number of completely different bioanalytes, from environmental samples to metabolic specimens and disease-related biomarkers^59–61^. Despite their many advantages over more traditional screening, sensing and diagnostic methods, the use of nanosensors are still not as widespread as it could be. In a quite provocative article, Vikesland^62^ suggest that although nanosensors present inherent capabilities of being highly sensitive, highly flexible, and have multiplex-prone functionalities, they are still not as consumer/operator-friendly as they should be in order to become more accessible. Many nanosensors depend on the use of uncommon electronic/optical devices to perform and cannot operate by using equipments that are immediately available in most diagnostic laboratories. Moreover, many such nanosensors may require specifically trained personnel either to operate the reading devices or to interpret the readouts^63–67^. Although the sensitivity and specificity of many published nanosensors are spectacular, those mentioned constraints might represent significant barriers to the sensors’ use, especially in developing and under-developed countries where they are frequently so desperately needed. Localized surface plasmon resonance-based nanosensors, on the other hand, are frequently based on optical readouts that can be obtained by simple UV–Vis spectrometry^68^. Refractive index changes induced by the binding of a molecule to the surface of functionalized metal nanoparticles result in spectral shifts that can be detected by a common spectrophotometer with UV–Vis capabilities, which are specifications frequently present in most ELISA plate readers. Because ELISA is a widespread diagnostic tool, this basic equipment is available in most diagnostic laboratories, even the humblest ones. Taking advantage of this potential for immediate applicability independently of the acquisition of specific reading devices, we developed a LSPR-based gold nanosensor for the differential immune diagnostic of DENV infections.

Despite the potential of the nanosensors described here, there are limitations and improvements that should be considered. One such limitation is the fact that spectra shifts are obtained as graphs in most spectrophotometers, and light wavelength alterations must be individually and manually compared in such devices, especially in the simplest ones. Incorporation of a software able to automatically obtain the final readouts would greatly improve the applicability of these sensing platforms and make them more operator-friendly. Another obvious question related to this platform is whether the use of recombinant proteins made in eukaryotic systems would improve the sensitivity and/or specificity of the sensors, as opposed to the bacteria-made proteins used here. It is possible that the use of proteins processed in eukaryotic cells would offer discontinuous antibody-recognizable epitopes that are not always present in proteins made in prokaryotic cells^46^. On the other hand, adding carbohydrates to the protein backbone could result is steric difficulties to link proteins to the gold surface. Such possibilities remain to be further evaluated.

## Methods

### Study design

The overall objective of the study was to develop and validate LSPR-based serologic nanosensors able to distinguish DENV-infected individuals from non-infected or other flavivirus-infected patients. This platform centers on the development of a fast and highly specific serologic tool based on electronic and optical properties of gold nanoparticles and their ability to quantify antigen–antibody pairs with different affinities. Gold nanorods were selected due to the plasticity of their LSPR spectrum. As a DENV diagnostic platform, the viral envelope protein was chosen as the biological component of the sensor based on the major humoral responses generated upon a DENV infection, regardless of immunoglobulin type. A well-established functionalization method—based on the covalent interaction between proteins and GNRs—was used to generate sensors with long-term stability and readout repeatability. Detection abilities were characterized using a range of monoclonal antibodies concentrations and highly diluted human sera. The specificity of the sensors was determined using sera samples from a well characterized cohort of flavivirus-infected patients, including ZIKA seropositive samples obtained during the 2015–2017 major ZIKV outbreak in Brazil. All spectra measurements were normalized to 1 and analyzed at the same wavelength in comparison to the negative controls to avoid unspecific spectral shifts. Measurements were analyzed using the OriginPro 8 software and all statistical analyses were performed using GraphPad Prism version 5.

### Recombinant DENV 1–4 envelope proteins expression and purification

The recombinant E-DENV proteins were expressed in *E. coli* using Brazilian circulating *Dengue virus* nucleic acid sequences available at the NCBI (GenBank) database (DENV1E: accession number GU131863, DENV2E: accession number GU131881, DENV3E: accession number JN697379, DENV4E: accession number KP188566). The coding genes were synthesized by Genone (Rio de Janeiro, RJ, Brazil) and synthetic genes were subcloned in pET-21a plasmid vectors. The cloned genes code for the 80% N-terminal portion of the DENV E proteins, amino acids 1–404, as the removal of the protein hydrophobic C-terminus has been shown to increase protein yield during its heterologous expression as well as its immunogenicity^69^. For protein purification, cells were disrupted (French Press Cell Disrupter, Thermo Electron Corporation) using a binding buffer under agitation and then homogenized. The recombinant proteins were purified by nickel affinity chromatography under denaturing conditions with HisTrap HP column employing an ÄKTAprime plus system (GE Healthcare, USA). Fractions were screened by SDS-PAGE, quantified by Bradford (BioRad, USA) and pools of the most productive samples were concentrated using VivaSpin filters (GE Healthcare, USA). The identity of the proteins was confirmed by western blot using anti-histidine (GE Healthcare, USA) and anti-dengue virus 1 + 2 + 3 + 4 antibodies (ab9202, Abcam, UK—according to the antibody’s datasheet this hybridoma antibody reacts with each dengue serotype in dot blot, ELISA and fluorescent antibody tests).

### Sera bank cohorts

For this work, we established two different sera banks. For the characterization of the first sera bank, when samples were serologically pre-tested, PRNT (Plaque reduction neutralization test) was employed as a confirmatory test. When a molecular diagnostic procedure (PCR) was used as a pre-test, samples were further confirmed through ELISA (see Suppl. Table 1). DENV-positive serum samples selected in this bank are originated from Southeastern Brazil outbreaks, from 2006 to 2014, a period that precedes ZIKV introduction in this geographic area. ZIKV-positive serum samples were obtained from the Minas Gerais State Reference Laboratory (FUNED/MG) and include sera from ZIKV-seropositive women that were infected during their pregnancy and/or from their children. Samples were previously tested by molecular and serological assays (see Suppl. Table 1). The second sera bank consists of an open cohort of less-characterized sera (evaluated only by serological analysis) from patients of different age range and sex, collected from 2015 to 2017 in two different states in Brazil, São Paulo and Minas Gerais (see Suppl. Table 2). Both sera banks were used throughout the study.

### Enzyme-linked immunosorbent assay.

The diagnostic potential of the generated recombinant proteins was evaluated by an in-house ELISA as described elsewhere^70^. Briefly, Nunc™ Maxisorp™ 96-well plates (Thermo Fisher Scientific Inc, USA) were individually coated with 200 ng per well of recombinant DENV1E, DENV2E, DENV3E or DENV4E diluted in carbonate buffer at pH 9.6 and incubated overnight at 4 °C. The plates were washed and incubated with the blocking solution at RT for 2 h. Diluted patient sera (1:50) were added to the wells and incubated at RT for 60 min. Plates were, then, incubated with anti-human IgG antibodies conjugated to horseradish peroxidase (Cell Signaling Technology, EUA), diluted 1:5,000 at RT for 60 min. The reaction was terminated with 100 μL sulfuric acid and the absorbance measured at 450 nm using an spectrophotometer (Asys/Hitech Expert plus, Eugendorf, Austria).

### GNR-DENV functionalization

Commercial Gold Nanorods [Part# A12-(10-780)-(CTAB)-(DI Water)], size 10 × 38 nm, were purchased from Nanopartz™ (US/Canada). GNRs were covalently functionalized with the DENV recombinant proteins through a carbodiimide-activated amidation reaction^23^ (see Fig. 2). Firstly, we obtained a saturation curve of the reduced α-lipoic acid re-suspended in ethanol with concentrations ranging from 1 to 40 mM in order to define the concentration necessary to replace the CTAB (Cetyltrimethylammonium bromide) layers on GNR surface. The α-lipoic acid (LA, Sigma Aldrich, USA) reduction in dihydrolipoic acid (DHLA) was performed following a Burst protocol as described elsewhere^25^. After choosing the appropriate concentration, 4 mM of DHLA was added to the gold nanorod solution (0.039 mg/mL) and the suspension was sonicated in an ultrasonic bath (UNIQUE model U5C1850, 154 W, 25 kHz) at 55 °C for 30 min followed by 2 h at 30 °C. GNRs were, then, centrifuged at 5600*g* for 10 min and suspended in aqueous solution. The GNR-DHLA complex was kept at 4 °C and protected from light exposure. The stability of the complex was evaluated during approximately 16 months by UV–Vis spectroscopy. For functionalization with the target proteins, the modified GNRs (GNR-DHLA) were re-dispersed in a 10 mM phosphate buffer containing 16 mM EDC (1-Ethyl-3-(3-Dimethylaminopropyl)carbodiimide, Thermo Fisher Scientific Inc, USA) and 4 mM sulfo-NHS (N-hydroxysulfosuccinimide, Thermo Fisher Scientific Inc, USA) for 30 min in an ice bath under sonication. After another centrifugation step, GNR-DHLA was blocked with poly(ethylene glycol)-thiolate (5kD mPEG-SH, 10^–4^ mM, from Nanocs Inc., USA) for 10 min in an ice bath under stirring. Finally, 100 µg of DENVE from each serotype (DENV1E, DENV2E, DENV3E and DENV4E) were individually added to the activated GNR solutions for 1 h in an ice bath under sonication. The individually obtained nanoconjugates were named GNR-DENV1E, GNR-DENV2E, GNR-DENV3E or GNR-DENV4E.

### Characterization of the GNR, GNR-DHLA and GNR-DENV nanoconjugates

#### Transmission electron microscopy

Transmission electron microscopy (TEM) was carried out on a 120 kV FEI Technai G2-12 (Spirit BioTwin) microscope. Samples were directly dripped onto a holey carbon film supported on a copper grid (400 mesh) (Pelco^®^) without any further processing.

#### UV–Vis-NIR absorbance

UV–Vis spectroscopy was performed on a Varioskan Flash spectral scanning multimode reader (Thermo Scientific) at wavelengths of 200–800 nm using a 96-well quartz microplate (Costar). Each sample was measured three times in all experiments and the spectrum was plotted from the resultant median.

#### Zeta potential (ζ)

Analyses of the Zeta potential were conducted on a Zetasizer Nano ZS90 analyzer from Malvern at an angle of 173° at room temperature. A laser beam set at 633 nm was choose to measure the electrophoretic mobility of particles using principles of dynamic light scattering. The Zetasizer analyzer calculates the zeta potential of the sample using the Smoluchowski equation: ξ = μη/ε, where ξ is the zeta potential, μ is the mobility, η is the viscosity of the solution, and ε is the dielectric constant of the solvent^26^.

### Evaluation of GNR-DENV biosensor efficiency and limit of detection

To evaluate the effectiveness of the interaction between nanosensors and antibodies, initial assays were carried out by using monoclonal anti-dengue virus 1 + 2 + 3 + 4 antibody (ab9202, Abcam, UK) and patients’ sera. To figure out the limit of detection of the GNR sensors, serial dilutions of anti-DENV antibodies, containing from 10 µg to 1 fg of total antibody mass, were added to the functionalized GNR solutions (GNR-DENV1E, GNR-DENV2E, GNR-DENV3E or GNR-DENV4E). After 30 min of incubation in ultrasonic bath at 4 °C, UV–Vis spectroscopy measurements were carried out using Multiskan GO spectrophotometer (Thermo Scientific). Assessments were conducted independently, during three consecutive days, with three replicates each. Data plot was based on the resultant median absorbance between replicates. To more precisely measure the biosensing event we evaluated plasmon shift in each curve through the X-axis intercept of the derivative of the Gaussian peak for every curve. In all figures depicting LSPR detection, the spectra absorbance and the derivative calculus were plotted side by side. Data analysis focused on red shifting measurements of the longitudinal peak, and 3 nm was considered the equipment’s maximum accuracy. To add confidence, shifts of less than 5 nm were not considered significant. The biosensors’ limit of detection was also tested in the same way employing DENV-positive human sera diluted up to 1:100,000. Shifts were considered in relation to sensors with no sera or sensors added with flavivirus-negative sera, as specified in each experiment or figure.

### Evaluation of the biosensors’ specificity

In order to evaluate the ability of the constructed biosensors in discriminating flavivirus-positive and negative patients, diluted sera samples were added to the sensors. Human sera that were negative for flaviviruses were used as controls. The positive and negative sera were individually pooled according to their characterization (DENV+, YFV+/DENV−, SLEV+/DENV−, ZIKV+/DENV−, Flavivirus-), diluted in 1:10,000 ratio, and added to individual sensors (GNR-DENV1E, GNR-DENV2E, GNR-DENV3E or GNR-DENV4E). Once again, we focused on the red shifting measurements of the longitudinal peak and shifts of less than 5 nm were not considered significant. We further evaluated the specificity of the constructed sensors by analyzing their ability to discriminate sera from patients that were infected by different DENV serotypes. Serotype-specific DENV-positive sera were characterized by PCR and PRNT tests, confirming the infective DENV serotype of each individual patient. Sera from each patient was diluted in 1:10,000 ratio and individually added to the individual sensors. Spectral shifts were measured as described above. Shifts were considered in relation to sensors with no sera or sensors added with flavivirus-negative sera, as specified in each experiment or figure.

### Statistical analysis

This experiment was analyzed as a classic diagnostic performance study, evaluating the concordance between a proposed test (the LSPR-nanosensor) and a reference standard test (in this case, the ELISA) for their ability to identify a target condition. The chosen method for statistical evaluation is based on the number of samples that are classified as true positive (TP), true negative (TN), false positive (FP), or false negative (FN) in a diagnostic assay. In order to evaluate the diagnostic potential of the recombinant proteins in a classic ELISA test and the LSPR-Nanosensors, we calculated Total Sensitivity (TSe), Total Specificity (TSp), accuracy (AC) and the positive and negative predictive values (PPV and NPV, respectively)^71^. Total Sensitivity (“positivity in disease”) refers to the proportion of subjects who have the target condition (reference standard positive) and render positive test results. Likewise, Total Specificity (“negativity in health”) is the proportion of subjects without the target condition and render negative test results. The term “total” refers to the fact that results obtained for the four nanosensors (GNR-DENV1E, GNR-DENV2E, GNR-DENV3E and GNR-DENV4E were compared to the performance of the four DENV proteins in the ELISA tests). AC is also known as “diagnostic accuracy” or “test efficiency”, and represents the overall proportion of correct test results. PPV refers to the probability by which a patient with a positive test result actually has the disease, and NPV refers to the probability by which a patient with a negative test result actually has no disease. A good screening test should have high PPV and NPV. The Kolmogorov–Smirnov method was used as normality test to evaluate the data distribution. The ANOVA test with Bonferroni correction as multiple hypothesis tests were used to compare groups with parametric distribution and Kruskal–Wallis test with Dunn’s correction was used to compare data with non-parametric distribution. Cutoff values were calculated by the average of true negative sera plus two-times standard deviation values (2SD). All statistical analyses were performed using GraphPad Prism version 5.

### Ethics statement

The use of human sera was approved by Ethical Committees from the Faculdade de Medicina de São José do Rio Preto, SP, (FAMERP), Fundação Ezequiel Dias (FUNED) and Universidade Federal de Minas Gerais (UFMG). Identities of sera donors were coded and not revealed to anyone involved in this study. All procedures followed ethical guidelines in accordance to national regulations. Samples were obtained in a cohort study with the written informed consent of the subjects. The study was approved by IRB (Comite de Etica em Pesquisa FAMERP) with the following approval codes: #32993014.0.0000.5415 and #02078812.8.0000.5415.

## Supplementary information

Supplementary Information.

## Data Availability

All data and materials are made publically available.
