# Supplementary material for: Nanosensors based on LSPR are able to serologically differentiate dengue from Zika infections
Source: Sci Rep. 2020 Jul 9;10:11302. doi: 10.1038/s41598-020-68357-9 (PMC7347616; doi:10.1038/s41598-020-68357-9)
Supplement: Supplementary file 1 — Supplementary Information. [file 41598_2020_68357_MOESM1_ESM.docx]

**NANOSENSORS BASED ON LSPR ARE ABLE TO SEROLOGICALLY DIFFERENTIATE DENGUE FROM ZIKA INFECTIONS**

Alice F. Versiani^1,6,#^, Estefânia M. N. Martins^2,6^, Lidia M. Andrade^3,6^, Laura Cox^1^, Glauco C. Pereira^4^, Edel F. Barbosa-Stancioli^1^, Mauricio L. Nogueira^5^, Luiz O. Ladeira^3,6^, Flávio G. da Fonseca^1,7^*.

**SUPPLEMENTARY MATERIALS:**


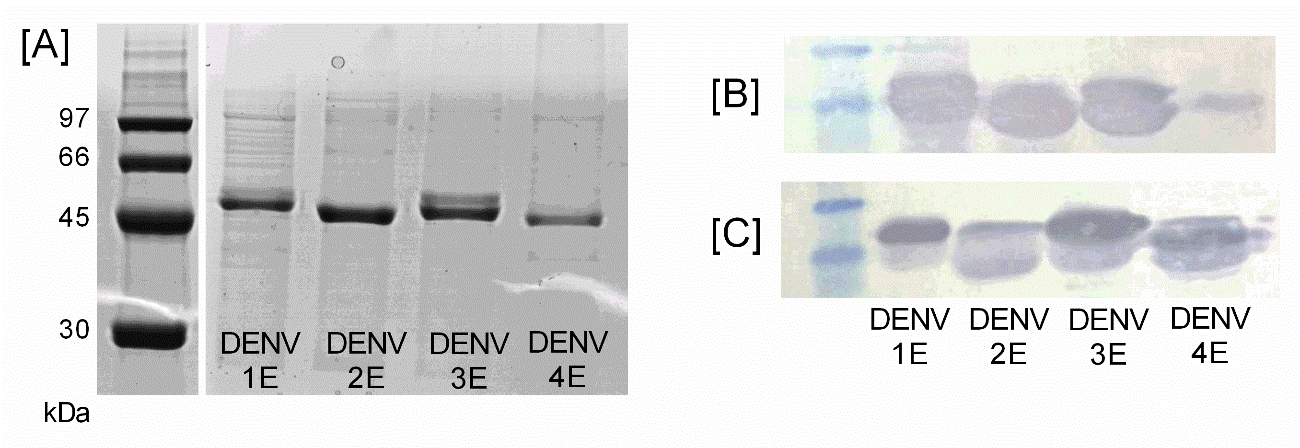


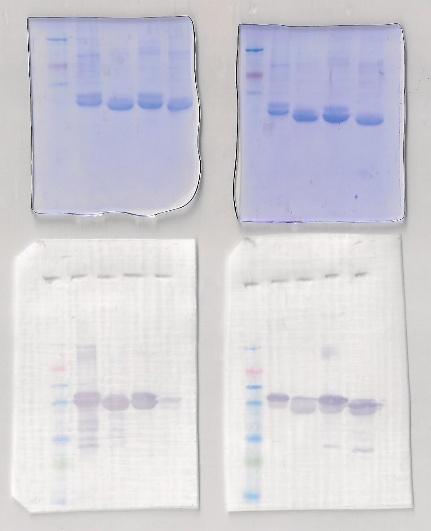


[D]


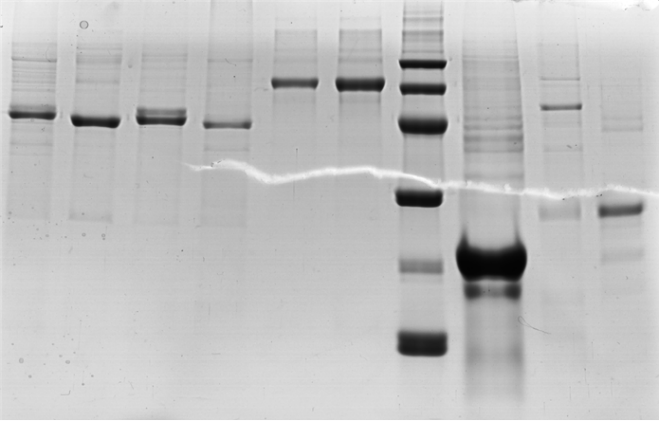



**Fig. S1***.* Recombinant DENV envelope proteins characterization. [A] SDS-PAGE. [B] Western blot with a monoclonal antibody anti-DENV. [C] Western blot with a monoclonal antibody anti-HIS. kDa: kilodaltons (molecular weight). Originals of gels and PVDF membranes are shown on [D] with a dashed line marking the image crops.

**Table S1.** Accurate sera bank composed of well-characterized patient serum for test validation.

| **Classification** | **Number of samples** | **Origin** | **Pre-test** | **Confirmatory test** |
| --- | --- | --- | --- | --- |
| **DENV1** | 20 | FAMERP/SP | PCR  Rapid test | ELISA  PRNT |
| **DENV2** | 20 |  |  |  |
| **DENV3** | 20 |  |  |  |
| **DENV4** | 20 |  |  |  |
| **SLEV** | 10 | Serro/MG | ELISA | PRNT |
| **YFV** | 4 | UFMG/MG | ELISA | PRNT |
| **ZIKV** | 20 | FUNED/MG | PCR | ELISA |
| **NEG** | 20 | FAMERP/SP | PCR  Rapid test | ELISA |

DENV: Dengue virus, SLEV: Saint Louis Encephalitis virus, YFV: Yellow Fever virus, ZIKV: Zika virus, NEG: Negative serum, FAMERP: Faculdade de Medicina de São Jose do Rio Preto from São Paulo state , Serro: city from Minas Gerais state, UFMG: Universidade Federal de Minas Gerais from Minas Gerais state, FUNED: Fundacao Ezequiel Dias, a Minas Gerais state reference laboratory for infectious diseases.

**Table S2.** Open sera bank composed of patient serum screened at public healthcare facility.

| **Classification** | **Number of samples** | **Origin** | **Pre-test** | **Confirmatory test** |
| --- | --- | --- | --- | --- |
| **DENV** | 35 | FUNED/MG | ELISA | ELISA |
| **ZIKV** | 64 | FUNED/MG | ELISA | ELISA |
| **NEG** | 52 | FUNED/MG | ELISA | ELISA |
| **NEG** | 40 | SERRO/MG | ELISA | ELISA |

DENV: Dengue virus, ZIKV: Zika virus, NEG: Negative serum, FAMERP: Faculdade de Medicina de São Jose do Rio Preto from São Paulo state , Serro: city from Minas Gerais state, UFMG: Universidade Federal de Minas Gerais from Minas Gerais state, FUNED: Fundacao Ezequiel Dias, a Minas Gerais state reference laboratory for infectious diseases.

**Table S3.** Diagnostic performance for the recombinant proteins DENV1E, DENV2E, DENV3E and DENV4E in the in-house ELISA test.

| **Parameters** | **DENV1E** | **DENV2E** | **DENV3E** | **DENV4E** |
| --- | --- | --- | --- | --- |
| Cutoff | 0.374 | 0.395 | 0.584 | 0.386 |
| **TSe (%)** | **80.2** | **59.5** | **71.6** | **69.1** |
| **TSp (%)** | **93.7** | **93.0** | **85.5** | **91.5** |
| PPV (%) | 87,8 | 82,5 | 74,4 | 82,4 |
| NPV (%) | 89,3 | 80,5 | 83,7 | 83,9 |
| AC (%) | 88,8 | 81,0 | 80,4 | 83,4 |
| TP | 65 | 47 | 58 | 56 |
| TN | 133 | 132 | 118 | 130 |
| FP | 9 | 10 | 20 | 12 |
| FN | 16 | 32 | 23 | 25 |

*TSe* total sensitivity, *TSp* total specificity, *PPV* positive predictive value, *NPV* negative predictive value, *AC* accuracy, *TP* true positive, *TN* true negative, *FP* false positive, *FN* false negative.





**Fig. S2.** UV-Vis-NIR follow-up after GNR-DHLA interaction. The GNRs were evaluated for six months after interaction with the DHLA. The DHLA capped GNRs were kept at 4-8°C protected from light.

**Table S4***.* LSPR-Nanosensor shift when tested with individual patient serum (DENV1 positive, DENV2 positive, DENV3E positive and DENV4 positive) and individual control serum (Negative serum and ZIKV-positive/DENV-negative serum).

| **Samples**  **Dilution of 1:100.000** | | **Shift (nm)** | | | |
| --- | --- | --- | --- | --- | --- |
|  |  | **Nanosensor**  **GNR-DENV1E** | **Nanosensor**  **GNR-DENV2E** | **Nanosensor**  **GNR-DENV3E** | **Nanosensor**  **GNR-DENV4E** |
| Negative sera | NS 12 | 3 nm** | 0 nm | 10 nm | 6 nm |
|  | NS23 | 2 nm** | 0 nm | 4 nm | 8 nm |
|  | NS 146 | 5 nm | 2 nm** | 0 nm | 6 nm |
|  | NS 1217 | 6 nm | 5 nm | 10 nm | 12 nm |
|  | NS 1319 | 0 nm | 3 nm** | 1 nm** | 0 nm |
|  | NS 1349 | 0 nm | 3 nm** | 0 nm | 0 nm |
| ZIKV+/DENV-  sera | ZV 37 | 0 nm | 4 nm | 10 nm | 3 nm** |
|  | ZV 51 | 0 nm | 0 nm | 11 nm | 4 nm |
|  | ZV 52 | 5 nm | 5 nm | 17 nm | 4 nm |
|  | ZV 83 | 1 nm** | 0 nm | 0 nm | Aggreg* |
|  | ZV 114 | 0 nm | 0 nm | 5 nm | 10 nm |
|  | ZV 127 | 0 nm | 0 nm | 7 nm | 9 nm |
| DENV1 positive sera | DV1 203 | **2 nm**** | 3 nm** | 0 nm | 0 nm |
|  | DV1 207 | **16 nm** | 0 nm | 0 nm | 0 nm |
|  | DV1 1903 | **23 nm** | 5 nm | 24 nm | 9 nm |
|  | DV1 2397 | **13 nm** | 0 nm | 0 nm | 0 nm |
| DENV2 positive sera | DV2 350 | 24 nm | **7 nm** | 8 nm | 0nm |
|  | DV2 547 | 0 nm | **10 nm** | 0 nm | 0 nm |
|  | DV2 2399 | 0 nm | **5 nm** | 0 nm | 0 nm |
|  | DV2 2403 | 0 nm | **9 nm** | 0 nm | 0 nm |
| DENV3 positive sera | DV3 364 | 4 nm | 0 nm | **15 nm** | 4 nm |
|  | DV3 445 | 0 nm | 0 nm | **15 nm** | 0 nm |
|  | DV3 525 | 0 nm | 0 nm | **16 nm** | 0 nm |
|  | DV3 1978 | 4 nm | 11 nm | **25 nm** | 0 nm |
| DENV4 positive sera | DV4 152 | 4 nm | 4 nm | 9 nm | **20 nm** |
|  | DV4 1901 | 1 nm** | 2 nm** | 10 nm | **16 nm** |
|  | DV4 1982 | 0 nm | 0 nm | 0 nm | **30 nm** |
|  | DV4 1998 | 0 nm | 0 nm | 0 nm | **31 nm** |

* Aggregated, ** negative shift considering the equipment accuracy.
